# Interventions to Address Environmental Metabolism-Disrupting Chemicals: Changing the Narrative to Empower Action to Restore Metabolic Health

**DOI:** 10.3389/fendo.2019.00033

**Published:** 2019-02-04

**Authors:** Robert M. Sargis, Jerrold J. Heindel, Vasantha Padmanabhan

**Affiliations:** ^1^Department of Medicine, University of Illinois at Chicago, Chicago, IL, United States; ^2^Program on Endocrine Disruption Strategies, Commonweal, Bolinas, CA, United States; ^3^Department of Pediatrics, University of Michigan, Ann Arbor, MI, United States

**Keywords:** diabetes, endocrine disruptor, non-alcoholic fatty liver disease, intervention, metabolism, obesity, metabolism-disrupting chemical

## Abstract

Metabolic disease rates have increased dramatically over the last four decades. Classic understanding of metabolic physiology has attributed these global trends to decreased physical activity and caloric excess; however, these traditional risk factors insufficiently explain the magnitude and rapidity of metabolic health deterioration. Recently, the novel contribution of environmental metabolism-disrupting chemicals (MDCs) to various metabolic diseases (including obesity, diabetes, and non-alcoholic fatty liver disease) is becoming recognized. As this burgeoning body of evidence has matured, various organic and inorganic pollutants of human and natural origin have emerged as metabolic disease risk factors based on population-level and experimental data. Recognition of these heretofore underappreciated metabolic stressors now mandates that efforts to mitigate the devastating consequences of metabolic disease include dedicated efforts to address environmental drivers of disease risk; however, there have not been adequate recommendations to reduce exposures or to mitigate the effects of exposures on disease outcomes. To address this knowledge gap and advance the clinical translation of MDC science, herein discussed are behaviors that increase exposures to MDCs, interventional studies to reduce those exposures, and small-scale clinical trials to reduce the body burden of MDCs. Also, we discuss evidence from cell-based and animal studies that provide insights into MDC mechanisms of action, the influence of modifiable dietary factors on MDC toxicity, and factors that modulate MDC transplacental carriage as well as their impact on metabolic homeostasis. A particular emphasis of this discussion is on critical developmental windows during which short-term MDC exposure can elicit long-term disruptions in metabolic health with potential inter- and transgenerational effects. While data gaps remain and further studies are needed, the current state of evidence regarding interventions to address MDC exposures illuminates approaches to address environmental drivers of metabolic disease risk. It is now incumbent on clinicians and public health agencies to incorporate this knowledge into comprehensive strategies to address the metabolic disease pandemic.

## Changing the Narrative of Environmental Health

The desire to live a healthy, disease-free life is universal; however, this goal is sadly unattainable for most of us. Diseases occur across the lifespan, often without warning. While patients' goals are to achieve a quick cure for their disease, chronic conditions requiring life-long therapy in which the clinical approach is to “manage” the disease often plague patients. While appropriate and timely treatment can stave off many of the complications of these chronic medical problems, the individual and societal costs of this treatment strategy are staggering. A better approach, indeed the holy grail for addressing the current plague of chronic disease, is to focus on disease prevention, which better ensures long term health with attendant economic benefits. Thus, what is needed is a narrative change that shifts the focus from management to prevention.

Such efforts, however, require an understanding of the factors driving chronic disease pathogenesis as well as validated strategies for addressing those risk factors. There is overwhelming evidence linking environmental factors such as chemicals, diet, stress, drugs, and lifestyle to the etiology of many chronic diseases. Thus, there is a desperate need to include improvements in our environment as part of comprehensive efforts to reduce the burden of chronic disease. To this end, this review focuses on one essential aspect of environmentally-driven disease, namely exposure to environmental toxicants and approaches to mitigate their impact on disease susceptibility and development. Specifically, we focus on one category of environmental pollutants, those that cause metabolic disease (metabolism-disrupting chemicals, MDCs). These MDCs represent a subset of environmental endocrine-disrupting chemicals (EDCs) that have been shown to disrupt metabolic physiology or are associated with metabolic disorders in cell-based, animal, or epidemiological studies (1). This discussion of MDCs provides valuable proof-of-principle for developing comprehensive approaches to reduce environmentally-mediated disease. Importantly, the approaches described herein have universal applicability for reducing the impact of myriad environmental chemicals on human health and disease.

This discussion comes at a crucial time. Recent analyses focusing on a subset of MDCs suggest that these toxicants are significant contributors to the societal burden of metabolic diseases and their attendant healthcare costs (2). Furthermore, these analyses likely underestimate the total contribution of MDCs to metabolic disease burden as they were restricted to a small subset of suspected MDCs and do not take into account potential additive or synergistic effects resulting from combinatorial exposures. In addition, many patients are increasingly aware of the potential role environmental toxicants play in disease development; however, it is clear that knowledge gaps exist regarding the potential health impacts of environmental toxicants in a given individual. Moreover, there is a lack of comprehensive clinical guidance to empower patients to address their environmental risk. Improving approaches to environmental health requires an ability to explain the dangers imposed by exposure, which may not become apparent for decades, against the (in)conveniences of lifestyle and policy changes necessary to reduce those exposures. We hope this review will contribute to changing the narrative by focusing the attention of scientists, physicians/health professionals, policy makers, and families on the available evidence to reduce disease-inducing exposures while also stimulating research to enhance our capacity to prevent the environmental contribution to the devastating individual and societal burden of metabolic disease.

## The Confluence of Environment and Metabolic Health

Over the last several decades, there has been a staggering increase in the prevalence of metabolic diseases across the globe. According to the World Health Organization (WHO), obesity rates have tripled since 1975 (3), and non-alcoholic fatty liver disease is now a leading cause of liver failure (4). Diabetes currently afflicts 9.2% of the global population with an estimated 629 million individuals predicted to suffer from the disease by the year 2045 (5). Perhaps even more concerning is the fact that these diseases are emerging at ever younger ages, transforming obesity, fatty liver disease, and diabetes from diseases of adulthood into common pediatric conditions (6–9). While it is without question that genetic susceptibility challenged by caloric excess and physical inactivity are central drivers of this epidemic, these factors insufficiently account for the rapidity and magnitude of the metabolic disease pandemic. Thus, identifying and addressing other contributors is essential for improving public health.

Endocrine disrupting chemicals (EDCs), defined as “an exogenous chemical, or mixtures of chemicals, that interfere with any aspect of hormone action” (10), are emerging as additional contributors to this pandemic. Approximately 1,000 EDCs have been identified (11). These chemicals have been linked to a variety of diseases, especially when the exposure occurs during development (10, 12, 13). In 2006 the term “obesogen” was coined by the Blumberg Laboratory (14) to describe a subclass of EDCs that cause obesity, a theretofore poorly appreciated consequence of environmental exposures. Data began to be assimilated showing how toxicants were also associated with other metabolic diseases, including diabetes (15), and soon a new word “diabesogen” was coined to describe a chemical that could induce type 2 diabetes and obesity (16). Over the ensuing years, it became clear that some chemicals could cause obesity, type 2 diabetes, or non-alcoholic fatty liver disease, while also leading to the metabolic syndrome (1, 17–19). In 2017, Heindel et al. proposed the term “Metabolism-Disrupting Chemicals” (MDCs) or “Metabolism Disruptors” for this subclass of EDCs to simplify the nomenclature (1). Thus, while obesogens are a subclass of MDCs, MDCs are a subclass of the more general EDCs; furthermore, these data suggest that MDCs as a class have a potentially broader role in the pathophysiology of many metabolic diseases.

Indeed, cellular, animal, and human evidence now implicates diverse classes of environmental toxicants as MDCs, including but not limited to bisphenol A (BPA), phthalates, chemical constituents of air pollution, antifouling agents, polychlorinated biphenyls (PCBs), various pesticides, perfluoroalkyl substances (PFAS), nicotine, and toxic metals (1, 18, 20, 21). Importantly, the rapidly advancing science in this area has moved from identifying associations to ascribing specific molecular mechanisms to the metabolism-disrupting properties of many of these toxicants (19, 22–24). While the strength of evidence linking any given chemical to metabolic dysfunction varies, the expansion and maturation of the field has solidified environmental exposures as relevant contributors to metabolic disease risk. Furthermore, as the field has earned greater attention, patients are increasingly concerned about how their environment impacts their health and are desperate for clinical guidance on how to reduce their exposure risk. Evidence is now beginning to coalesce that will empower physicians and public health officials to translate MDC science into action to improve health.

## Windows of Susceptibility to MDCs

**Figure 1:**
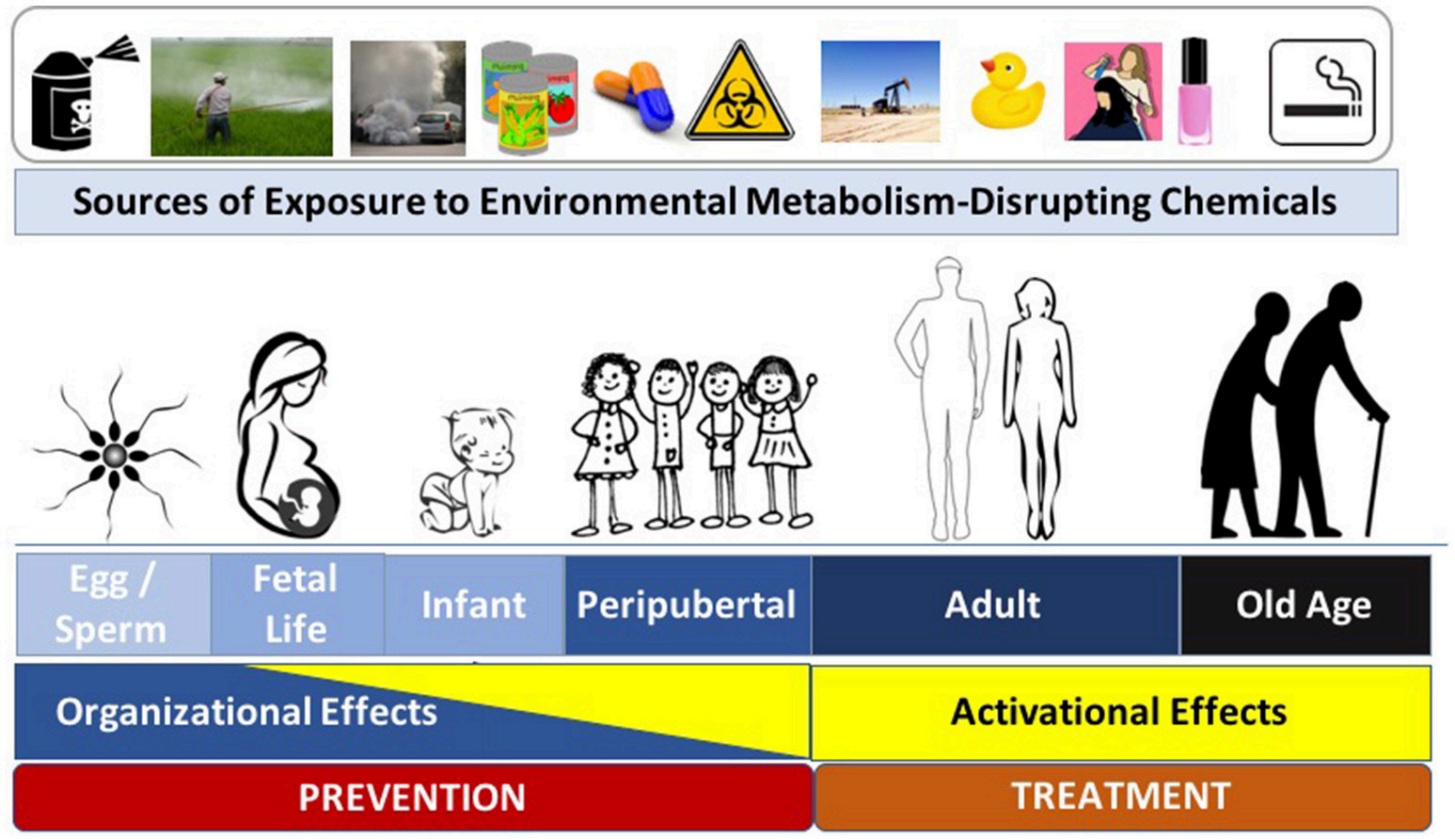
Metabolism-Disrupting Chemicals (MDCs) across the Lifespan. Exposure to MDCs can exert adverse effects across the lifespan. Later in life the effects of MDCs are principally activational; however, early in life, MDCs can exert organizational effects that program increased long-term risk to metabolic diseases. Interventions to prevent adverse metabolic effects from environmental toxicants include efforts aimed at disease prevention (to principally address potential organizational effects) and disease treatment (to principally address activational effects). Images from pixabay.com and openclipart.org.

To effectively focus on disease prevention via specific interventions, it is essential to understand the most sensitive times during which interventions are likely to have the most significant impact. Endocrine and metabolic signaling exert activational effects across the lifespan; during critical developmental windows, however, these signaling events also exert organizational effects that lead to permanent changes in tissue assembly. While metabolic regulation is sensitive to activational perturbations by MDCs across the lifespan, an emerging body of literature has specifically implicated MDC exposures during critical organizational windows of development in the pathogenesis of metabolic disease later in life (Figure 1). Known as the Developmental Origins of Health and Disease (DOHaD), this field has expanded from nutritional stressors to those imposed by toxicant exposures (13, 25, 26). While programming events occurring during development are likely essential for adapting to the external milieu, this process of “adaptation” may be corrupted when developmental programs and the external milieu are mismatched, creating a disease-promoting interaction between intrinsic and extrinsic risk factors (27). Indeed, exposure to several MDCs across developmental windows in early life (e.g., *in utero* and early postnatal [reviewed in *ref*. (1)]) are now implicated in the misprogramming of metabolic homeostasis. While the most sensitive window of exposure is during fetal development and the first years of life (i.e., the periods during which tissues are organizing), other sensitive periods, including preconception, pregnancy, and peripuberty, might also be sensitive to the deleterious effects of MDCs (28–30). Perhaps most disturbing are data from animal models showing that exposure to MDCs during development results in disease susceptibility that can be transmitted across generations (31, 32). Thus, reducing developmental exposures and antagonizing MDC effects are of paramount importance for preserving metabolic health across generations.

While the underlying mechanisms responsible for the sensitivity of a particular developmental window to the adverse effects of MDCs remain incompletely understood, epigenetic alterations are a likely culprit (13). Thus, mitigating exposures during early windows of susceptibility that induce epigenetic modifications is expected to have the most significant impact for preventing diseases arising from MDC exposure. It is clear, however, that eliminating exposures across the lifespan will also be essential to reduce activational effects of EDCs to lessen the severity of metabolic disease phenotypes. Thus, strategies to comprehensively address the impact of environmental toxicants on metabolic health must eliminate exposures that induce organizational effects to prevent disease as well as activational effects to lessen the severity of metabolic dysfunction.

## Strategies to Mitigate the Impact of Environmental MDCs

It is clear that reductions in the industrial production, use, and environmental dissemination of MDCs are an essential part of any strategy to limit the impact of this modifiable risk factor. However, additional approaches are required to bridge the gaps created by delays in the adoption of “green” chemistry or recalcitrance in moving to a “first do no harm” approach to economic development. There are three general categories of interventions necessary to protect human metabolic health: (1) strategies to empower individuals to reduce their exposures to MDCs; (2) interventions to reduce the burden of persistent pollutants that bioaccumulate; and (3) therapeutic/dietary approaches to antagonize the deleterious effects of exposures. Current evidence suggests that paradigmatic approaches are evolving in each of these core areas. This new information should provide the impetus to include environmental management strategies as essential pillars of clinical care plans to comprehensively address the individual and societal burden of metabolic diseases. This is a vital step as most clinical practice guidelines are entirely blind to the role of environmental toxicants as mediators of metabolic risk (33–35). Importantly, efforts to incorporate environmental toxicology into clinical practice are not meant to suggest an usurpation of lifestyle interventions to address metabolic disease risk; rather, we propose adaptations to comprehensive frameworks to address the pandemic of metabolic disease that include adjunctive initiatives to address environmental contributors to disease risk.

### Reducing Exposures to MDCs

There are two ways to minimize exposure to MDCs: (1) reduce actual exposure to the chemicals themselves, or (2) reduce their post-exposure accumulation in the body. Understanding the sources of human exposure is key to clinical guidance to reduce exposure to MDCs. There are a variety of resources available that provide information on the origins of MDCs and how to limit contact with them. The Endocrine Disruptor Exchange (TEDx) (11), the Environmental Working Group (36), and the new Because Health (37) websites offer excellent examples of exposure reduction strategies. Information on air quality is also publicly available at AirNow.gov (38). There are also mobile apps to help reduce exposures and help find toxin-free food, cosmetics, and household products as well as to gain knowledge about air quality conditions (39–45). In addition, we have recently developed a concise Healthcare Provider Guide to empower practitioners to discuss exposure reduction strategies with their patients (46). The American Academy of Pediatrics (47) provides further guidance. Table 1 shows examples of prudent measures that can reduce exposures to MDCs. These measures are relevant across the lifespan from fetal life through childhood, puberty, adulthood, and aging.

**Table 1:** Interventions and resources to address exposures to metabolism-disrupting chemicals.

Personal care and hygiene	Wash your hands regularly using soaps without fragrances and antibiotics. Ensure you have clean hands before preparing and eating food. Minimize handling of receipts and thermal paper. Read labels and avoid products that contain phthalates and parabens. Avoid use of phthalate- and BPA-containing products; recognize that “phthalate-free” and “BPA-free” products may contain other replacement chemicals of concern. Avoid fragrances and opt for cosmetics labeled as “no synthetic fragrance,” “scented only with essential oils,” or “phthalate-free.” Avoid all cosmetics containing lead
Children	Encourage your local school council to reduce school bus emissions, including idling. Avoid hand-me-down plastic toys. Utilize glass alternatives for infant formula bottles. Recognize toys that are labeled “BPA-free” may contain other replacement chemicals of concern
During pregnancy	Ensure adequate intake of calcium, iron, and iodine. Consult guides on the safe intake of fish and seafood
Food and beverage	Eat a diversified diet with plenty of variety. Eat fresh and frozen foods, and reduce consumption of canned and processed foods. Prepare more meals at home with an emphasis on fresh ingredients. Wash fruits and vegetables before consuming them. If possible, purchase organic produce, meat, and dairy products. Choose foods grown and raised locally. Consider using a water filter. This is especially important for those using well water in areas in which arsenic contaminates groundwater as well as for those living in old houses with lead pipes. Store food in glass, stainless steel, or porcelain whenever possible, especially for hot liquids and foods. Avoid plastic containers, especially those designated #3, #6, and #7. Don't microwave foods and beverages in plastic containers. Trim fat from meat and the skin from fish. Cook meat and fish on a rack to let them drain. Consult local guidance regarding which sport fish are safe to consume. Eliminate consumption of sugar-sweetened beverages. Eat a diet that is high in fiber
Exercise and activity	Exercise! But choose times and places with better air quality. For instance, avoid exercise in high traffic areas if possible. Opt for routes away from busy roads. Avoid outdoor activities when air pollution levels are high
At home	Forbid smoking indoors. Do not burn trash. Using a damp cloth, regularly clean your floors and remove dust. Replace old fluorescent bulbs and deteriorating construction materials from older buildings. For those using well water supplied by a submersible pump, if you notice an oily film or fuel odor in your water, determine whether the pump has failed and replace it if necessary. Contact your local Department of Public Health for information on how to clean the well. Choose electrical appliances to limit indoor air pollution. Opt for paints that are low in volatile organic chemicals (VOCs). Limit use of household chemicals, including cleaning supplies, pesticides, and solvents
In the garden	Plant trees and preserve forests to filter air and reduce the “heat island effect”. Plant native species of plants and trees. Do not burn leaf litter and wood. Use hand-powered or electric lawn care equipment and eliminate use of gas-powered equipment. Eliminate use of all pesticides
Getting around	Use public transit whenever possible. Choose travel times and routes that limit idling. Walk or bicycle while using safe routes that limit exposure to air pollution. Avoid places that allow smoking
Advocate	Encourage funding for public transportation options as well as safe bicycling paths. Advocate for sustainable development that maximizes energy efficiency, preserves natural spaces, and encourages walkability. Encourage the development of municipal codes that mandate the use of green roofs, cladding buildings in plants such as ivy, and planting trees. Demand energy from renewable sources and infrastructure to support electric vehicles. Promote efforts to expand walking and bicycle paths. Encourage efforts to make public spaces tobacco-free, including restaurants and bars. Demand that municipalities, park districts, and golf courses eliminate the use of pesticides. Advocate for federal legislation to improve labeling of products so that consumers are adequately informed of their exposures
Sources and additional resources to identify and reduce exposures	The Endocrine Disruptor Exchange (TEDx) [https://endocrinedisruption.org] Environmental Work Group (EWG) [https://www.ewg.org] EWG's Skin Deep Guide to Cosmetics [https://www.ewg.org/skindeep/] EWG's Guide to Sunscreens [https://www.ewg.org/sunscreen/] Because Health [https://www.becausehealth.org] AirNow [https://www.airnow.gov] American Academy of Pediatrics [https://www.aap.org/en-us/Pages/Default.aspx] American College of Obstetrics and Gynecology [https://www.acog.org/Clinical-Guidance-and-Publications/Committee-Opinions/Committee-on-Health-Care-for-Underserved-Women/Exposure-to-Toxic-Environmental-Agents] Ruiz et al. *Diabetes Care*, 2018 [(46) as well as supplement and references therein]

To understand which interventions are likely to reduce exposures, it is instructive to examine those practices that increase contact with MDCs. Indeed, data from several studies demonstrate that behavioral choices impact MDC exposure. For example, phthalate levels are positively correlated with consumption of fast food (48, 49). Use of polycarbonate water bottles increases BPA levels (50), as does canned soup consumption (51). Because phthalates and BPA are non-persistent, individuals should recognize and limit their contact with potential sources in order to lower their burden of exposure.

Indeed, several clinical studies have attempted to test this supposition. In an intervention among Latina girls that focused on reducing exposure to personal care products, investigators showed reductions in mono-ethyl phthalate, methyl and propyl parabens, triclosan, and benzophenone (52). Similarly, restricting diets to foods with limited packaging decreased levels of BPA and diethylhexyl phthalate (DEHP) (53). Furthermore, a study in Taiwanese children showed that attention to handwashing coupled with reducing consumption of beverages from plastic cups decreased phthalate levels (54). These data support the conjecture that behavioral change can lower levels of non-persistent toxicants, including those linked to metabolic dysfunction. Such approaches to reduce MDC contact can, therefore, be employed across the lifespan, especially in pregnancy during which there is the potential to impact the metabolic health of multiple generations.

Air pollution is another significant source of MDCs. For example, residents of rural Michigan who were exposed to urban air for only 4–5 hours daily for 5 days exhibited an increase in the homeostatic model assessment of insulin resistance (HOMA-IR) for each 10 μg/m^3^ increase in particulate matter 2.5 μm in size or smaller (PM_2.5_) (55). Among individuals with the metabolic syndrome living in Beijing, China, increases in PM_2.5_ and black carbon correlated with increases in insulin resistance (56). In a small study of individuals with preexisting diabetes, vascular dynamics were shown to be adversely affected by increases in PM_2.5_ (57). These data suggest that the relationship between metabolic function and air quality are, in fact, dynamic. As such, efforts to improve air quality are likely to have salutary effects on population-level metabolic health. On an individual level, avoidance of exposures (e.g., by avoiding heavily-traveled streets during commutes or while exercising) may be beneficial. Similarly, avoiding outdoor activities during periods of poor air quality are also likely necessary, as is cessation of practices that worsen local air quality such as burning organic matter, using gasoline-powered lawn care equipment, smoking indoors, and using household chemicals. Patients can be empowered to consider air quality when making choices about outdoor activities through publicly-available websites such as AirNow (38) (Table 1).

Despite such encouraging avenues to reduce exposure by behavioral changes, some critical questions remain regarding the scalability and persistence of these types of interventions and the state of our knowledge regarding the impact of MDC exposure during different developmental windows. In a 3-day study of pregnant women, provision of a diet consisting mostly of fresh, organic foods for 3 days did not lower phthalate levels, perhaps due to the short study time (58). Furthermore, additional concerns regarding the efficacy of behavioral interventions were dramatically raised by a study that sought to lower phthalate levels through the substitution of all meals with organic alternatives. This study did not only fail to reduce phthalate levels, but levels of DEHP *rose* markedly (59). Further, examination revealed a concentrated source of phthalates in the organic alternatives: ground coriander and milk. This study underscores one of the central challenges in providing individuals with clinical guidance on exposure reduction strategies. Current laws regulating the labeling of foods, personal care products, and other goods are woefully inadequate when it comes to providing consumers with knowledge about the chemicals that they contain. In addition to closing these gaps in exposure knowledge, further studies are also required to unequivocally show that interventions that lower exposure levels also meaningfully improve metabolic health.

### Addressing MDC Exposure During Critical Developmental Windows

Developmental periods during early life are especially sensitive windows during which exposure to MDCs increases long-term disease susceptibility. Thus, it is important to prioritize exposure reduction during pregnancy and early childhood, the periods during which metabolic tissues are organizing and therefore exquisitely sensitive to misprogramming events that augment later life metabolic disease risk. Many of the same methods that are useful to reduce exposures in adults are also helpful during pregnancy. In addition, since misprogramming may result from altered epigenetic regulation, which is potentially heritable, interventions that antagonize toxicant-induced epigenetic changes may represent viable approaches for mitigating the developmental origins of metabolic dysfunction induced by MDCs across generations. Indeed, there are data showing proof-of-principle for strategies that can prevent epigenetic misprogramming. In seminal work, the MDC BPA was shown to shift coat color in A^vy^ mice via DNA hypomethylation; diets supplemented with methyl donors, including betaine, choline, folic acid, and vitamin B12 antagonized this effect (60). Interestingly, this impact of methyl donors may be relevant in humans as well. In a cross-sectional study of couples undergoing assisted reproductive interventions, high maternal folate intake attenuated the negative association between BPA levels and *in vitro* fertilization success (61). Whether these benefits arise from changes in DNA methylation or are dependent on other factors requires further study, but taken together, these data suggest that interventions to support one-carbon metabolism may modulate some of the adverse effects of some developmental MDC exposures.

This premise is, however, not a clear panacea. In a mouse model of developmental arsenic exposure, folate supplementation lowered the burden of arsenic in maternal livers but did not prevent toxicant-induced reductions in fetal body weight and increases in hepatic S-adenosylmethionine and S-adenosylhomocysteine levels (62). Moreover, this study showed that coordinate exposure to folate and arsenic led to marked changes in DNA methylation, including adverse effects on genes regulating fetal development, suggesting that combined high folate intake in the context of arsenic exposure may *augment* fetal risk (62). In contrast, maternal folate and B12 supplementation reversed the effects of prenatal arsenic exposure on fasting hyperglycemia and insulin resistance in the male offspring of dams maintained on a diet adequate for folate and vitamin B12 (63). Importantly, there were no global hepatic DNA methylation changes associated with the effects of arsenic on glucose homeostasis (63). These data raise important questions about the potential to prevent adverse effects of MDCs on metabolic programming by modulating the epigenetic machinery. While the possibility to meaningfully intervene exists in some contexts, it remains unclear how specific the intervention is to the insult. In other words, the ability to target epigenetic interventions to the defects induced by MDC exposures may lack the specificity to achieve the desired effect. Moreover, in the worst case, as with folate supplementation with arsenic, the intervention may worsen outcomes. Nonetheless, as knowledge of epigenetic programming expands and specific alterations in epigenetic marks and gene expression are defined as biomarkers of exposure, it is likely that this area will become a focus of future intervention strategies.

### Therapeutic Approaches to Antagonize the Deleterious Effects of MDCs

As knowledge and understanding of MDCs has evolved, an emerging field of interest focuses on reducing adverse MDC effects by directly targeting their mechanisms of toxicity. To date, there are a limited number of studies in this area to suggest that some interventions may mitigate the adverse metabolic impact of MDCs. For example, the natural plant phenol resveratrol has been shown to antagonize PCB-induced impairments in adipocytic insulin signaling *in vitro* and *in vivo* with attendant improvements in systemic glucose tolerance and insulin sensitivity (64). *N*-acetylcysteine (NAC) is an FDA-approved medication used to treat acetaminophen (paracetamol) toxicity, to address respiratory secretions for those with lung diseases, as well as in other conditions. The benefits of NAC in protecting against acetaminophen-induced hepatic injury are thought to arise through elevations in the antioxidant glutathione. In rat models, NAC has been shown to reduce PCB-induced hepatic steatosis (65) and to prevent glucose intolerance resulting from acute exposure to arsenic (66). NAC has also been shown to antagonize arsenic-induced alterations in glucose homeostasis in mice, potentially by protecting pancreatic β-cells (67). When used in conjunction with monoisoamyl dimercaptosuccinic acid (DMSA), NAC restored liver glutathione levels and protected against chronic arsenic poisoning in guinea pigs (68). Pretreatment with NAC also attenuated arsenic-induced hepatic injury and mitochondrial dysfunction (69). In another study of combinatorial interventions, co-treatment with NAC and *meso*-2,3-DMSA reduced hepatic oxidative damage to a greater extent than either agent alone (70). In rats, NAC protected against arsenic-induced liver toxicity, and coadministration of zinc potentiated this effect (71). In a separate study, however, zinc was shown to have limited capacity to mitigate or prevent PCB-induced liver toxicity in rats despite known disruptions in zinc metabolism stemming from PCB exposure (72). These data provide tantalizing evidence that interventions during adulthood have the capacity to mitigate the deleterious effects of some MDCs on tissues regulating metabolic homesostasis by targeting the mechanisms and downstream effectors of MDC action. However, further data are required to better understand how applicable these interventions are to other MDCs as well as whether they are safe and effective interventions in human populations, especially pregnant women and children.

There are also approaches that focus on pathways common to many environmental chemicals, namely oxidative stress and/or inflammation. A “healthy” diet can itself reduce the risk of chronic inflammation, thus reducing the effects of some MDCs (73). Foods rich in fruits and vegetables, green tea, and omega-3 fatty acids can reduce inflammation and oxidative stress via the upregulation of antioxidant enzymes; this can reduce MDC effects, including cardiovascular toxicity (74–76). For example, broccoli sprouts contain chemicals that generate sulforaphane, which induces antioxidant enzymes; consumption of a broccoli sprout beverage providing 600 μmol glucoraphanin and 40 μmol sulforaphane daily for 12 weeks increased excretion of conjugates of the toxicants benzene and acrolein (77).

Several vitamin and mineral deficiencies during pregnancy have been shown to promote metabolic dysfunction in offspring later in life, which could interact with MDCs to increase disease manifestation and severity. These include deficiencies in vitamin B12 (78), chromium (79), and zinc (80). The similarity of outcomes resulting from such deficiencies and MDC exposures raise several fundamental questions. First, do MDCs that alter metabolic programming elicit their effects in whole or in part by altering vitamin and mineral metabolism? Second, does repletion or supplementation with these vitamins and minerals antagonize or rescue the adverse impact of MDCs on metabolic function? Understanding the commonalities and differences across classes of developmental stressors that potentiate long-term metabolic disease risk are likely to offer practitioners insights into interventions to protect the health of pregnant women and children. While this area of nutritional intervention is still in its infancy, these data suggest the potential for dietary interventions to be an effective and safe approach to reducing the toxicities of MDCs as well as other EDCs.

Growing evidence points to EDCs such as arsenic, lead, and nanoparticles affecting the gut microbiome (81–83). In turn, gut microbiota may modulate environmental chemical toxicity (84, 85). For instance, a study conducted in mice found that exposure to BPA from periconception through weaning resulted in sex-specific and generational differences in the gut microbiome and metabolic pathways related to metabolic dysfunction (86). Considering that gut microbiota play a role in metabolizing MDCs and have the potential to alter their toxicodynamics as well as their detrimental effects, the therapeutic utility of microbiome manipulation via probiotic supplementation is a fertile avenue for investigating future therapeutic interventions.

### Effective Interventions in the Context of Non-MDC Developmental Stressors

In the search for effective interventions for developmental MDC exposures, manipulations that antagonize the adverse metabolic effects of other types of environmental stressors may be helpful. For example, inducing stress in dams during the last week of pregnancy by repeated restraint results in alterations in hippocampal glucocorticoid receptors in adult offspring; blocking maternal corticosterone production blunted this effect (87). In a study of rats, early post-natal handling was able to mitigate some of the adverse effects of prenatal stress on programming of the hypothalamic-pituitary-adrenal (HPA) axis (88). Because HPA hyperactivation promotes metabolic dysfunction due to excessive production and/or action of glucocorticoids, these data suggest that *in utero* or early post-natal interventions to address HPA programming may have beneficial effects on metabolism. Although the extent to which HPA programming contributes to the action of MDCs remains to be clarified, the centrality of this endocrine axis to metabolic fate suggests that interventions that prevent the hyperactivation of HPA signaling may help antagonize metabolic disruptions induced by some MDCs.

*In utero* nutritional status has been linked to metabolic dysfunction later in life. A study using rats selectively bred to develop diet-induced obesity showed that rearing pups in large litters vs. normal litters (16 vs. 10 pups/dam) resulted in an attenuation of diet-induced obesity with attendant changes in leptin signaling (89). In another intriguing study, provision of a running wheel to rats sensitive to diet-induced obesity at 36 days of age resulted in reduced adiposity and increased thermogenesis relative to sedentary controls; furthermore, only 3 weeks of exercise was sufficient to reduce long-term weight gain (90). These data suggest that, at least in rats, early post-natal interventions may reprogram some metabolic parameters to favor *improved* metabolic health. How these interventions may work in negating the impact of developmental MDC exposures in humans and especially pregnant women and children remains to be explored.

### Reducing the Legacy of Persistent Pollutants

Among environmental toxicants linked to metabolic dysfunction, some of the best characterized are persistent organic pollutants (POPs). These include such legacy contaminants as polychlorinated biphenyls (PCBs) and dioxins as well as organochlorine pesticides such as dichlorodiphenyltrichloroethane (DDT). While production of many of these toxicants has been banned or significantly restricted, their biological and environmental persistence ensures they are, and will continue to be, relevant from an environmental perspective for many years to come. Persistent pollutants are passed down from generation to generation across the placenta or through lactation (91, 92), which underscores their continued importance. Thus, interventions are required to reduce the body burden of these toxicants to avoid transmitting their risk to future generations.

One approach to eliminate POPs leverages the fact that many of these toxicants undergo enterohepatic circulation. The non-absorbable fat olestra (Olean™) is not a substrate for pancreatic lipases; as such, it is excreted unchanged in feces. It was commercialized in the 1990s in a variety of processed foods as a means to provide the qualities of fatty foods without the attendant caloric content. In high quantities, however, olestra induces steatorrhea. For this reason, as well the loss of fat soluble vitamins due to reduced fat absorption, olestra was removed from the market. While deleterious in the context of vitamin metabolism, the capacity of olestra to facilitate passage of fat soluble molecules from the gastrointestinal tract has the potential to clear MDCs that undergo enterohepatic circulation. The capacity of olestra to remove POPs was examined in a series of small studies. In two patients with chloracne (eruption of blackheads, cysts, and pustules resulting from over-exposure to certain halogenated aromatic compounds), olestra was shown to accelerate the fecal excretion of 2,3,7,8-tetrachlorodibenzo-*p*-dioxin (TCDD) (93). In a case of organochlorine toxicity, namely contamination with the PCB mixture Arochlor 1254, treatment for 2 years with olestra (~16 g/day) led to weight loss with concordant improvements in the subject's diabetes and dyslipidemia (94). In a randomized, year-long trial of residents with elevated serum PCB levels in Anniston, Alabama, elimination of 37 coplanar PCBs was accelerated in the olestra group (15 g/day), but not among those receiving vegetable oil (95). The context of the intervention may be important, however. Weight loss increases circulating levels of POPs (96–100). In one study, replacement of 33% of dietary fat with olestra reduced β-hexachlorocyclohexane concentrations, but did not attenuate the expected weight loss-induced rise in other organochlorines (101). While not necessarily conclusive, these data provide critical evidence that approaches to promote excretion of lipophilic compounds may be useful to reduce levels of POPs linked to metabolic dysfunction. In another study, treatment of 15 healthy women with 1,000 mg per day of vitamin C for 2 months also reduced levels of six PCBs and two organochlorines pesticides; however, levels of polybrominated diphenyl ethers (PBDEs) were unaffected (102).

While these studies demonstrate the potential to lower lipophilic MDC levels, several issues remain. Many of these studies were either small or case reports. Furthermore, except for one case study, the metabolic impact of actively reducing POP levels has not been shown. More extensive studies examining MDC elimination with metabolic phenotyping will be necessary for supporting such interventions. A second concern is that the side effects that led to olestra's removal from the commercial marketplace are likely to lead to poor patient adherence as part of any clinical intervention. As such, alternative approaches with a better risk profile are needed. Indeed, reasonable FDA-approved alternatives may already exist. Bile acid sequestrants are used to lower plasma cholesterol levels by facilitating fecal excretion of bile acids, which leads to the upregulation of the low-density lipoprotein receptor (LDL-R) in the liver and consequential clearance of LDL and its attendant cholesterol from the circulation. In addition to removing bile acids, these pharmaceutical agents also facilitate excretion of other molecules from the body. For example, the bile acid sequestrant cholestyramine is used as an adjunctive therapy in the treatment of thyrotoxicosis due to its ability to disrupt the enterohepatic circulation of thyroid hormone by facilitating its fecal excretion (103). Interestingly, in work published over 25 years ago, cholestyramine was shown to augment fecal excretion of PCBs in rats (104). In a clinical study of individuals from the Yucheng Cohort, combination treatment with cholestyramine and rice bran fiber augmented excretion of PCBs and polychlorinated dibenzofuran with some inter-individual variation in effectiveness (105). The question of whether cholestyramine can augment excretion of stored POPs in less highly exposed individuals warrants testing. It is also essential to determine whether this approach is broadly applicable to other lipophilic POPs. The fact that the bile acid sequestrant colesevelam has been shown to improve glucose homeostasis (106) underscores the importance of such a study. While this has been presumed to result from alterations in bile acid signaling, removal of glucose-raising POPs remains a plausible additional mechanism for colesevelam's beneficial metabolic effects.

Pharmacological agents that inhibit fat absorption may also promote excretion of POPs similar to olestra. Orlistat (Xenical™, Alli™), an inhibitor of pancreatic lipase, is FDA-approved for the treatment of obesity. While part of orlistat's weight loss effect is likely a consequence of behavioral changes to avoid fat consumption to limit adverse gastrointestinal side effects from fat malabsorption (e.g., steatorrhea, flatulence, and fecal incontinence), use of this agent on a low fat diet may yet facilitate clearance of fat soluble POPs by increasing fecal fat loss and is worth investigating. Of course, the risks of malabsorption, including the loss of fat soluble vitamins and essential fatty acids will need to be taken into account for any approach employing fecal fat loss.

One area of longstanding interest for removing PCBs and other legacy chemicals is dietary fiber. For instance, the dietary fibers pectin and konjac mannan attenuated the PCB-induced elevations in serum protein, high density lipoprotein-cholesterol, triglycerides, and liver lipids in rats (107). In another study, a chitosan-supplemented diet increased fecal excretion of PCBs when compared to fiber-free diets (108). Interestingly, compared to diets with water-insoluble fiber, fermentable fibers (polydextrose, indigestible dextrin, and soy polysaccharides) increased urinary PCB excretion (108). In a small study of nine Japanese couples followed for 2 years, consumption of fermented brown rice with *Aspergillus oryzae* (7.5–10.5 g immediately after each meal for 2 years) augmented elimination of polychlorinated dibenzo-*p*-dioxins and polychlorinated dibenzofurans (109).

The precise impact of fiber on MDCs are likely influenced by both the specific fiber as well as the MDC of interest. In an *in vitro* study, dietary fibers were shown to bind several polycyclic hydrocarbons; however, rice bran fiber and pulp lignin produced stronger effects than other types of fiber, including corn, wheat bran, and spinach among others (110). Indeed, in rat studies, wheat bran has complicated effects on PCB absorption and excretion. For example, compared to placebo, the addition of wheat bran to the diet augmented fecal excretion of dietary PCBs. Furthermore, switching from a control diet to a wheat bran-enriched diet enhanced excretion of previously absorbed PCBs; however, the impact of the wheat bran diet did not significantly lower adipose and hepatic PCB levels (111). In a follow-up study, wheat bran-enriched diets relative to a cellulose-based placebo during PCB exposure enhanced fecal excretion of PCBs; however, upon cessation of PCB exposure, wheat bran had a minimal impact on excretion of accumulated PCBs (112). Moreover, the effect of wheat bran on total body PCB accumulation and retention was determined to be minimal in both studies (111, 112). It appears that the chemical structure of the toxicant influences its ability to bind to fiber (110), suggesting that the potential benefit of dietary fiber interventions may be dependent on the specific MDC studied. Importantly, there do not appear to be any studies that have examined the impact of dietary fiber on non-persistent pollutants such as bisphenols and phthalates.

### Weight Loss as Opportunity and Threat

Weight loss is a frequent intervention to reduce the risk of metabolic disease; however, weight loss has paradoxical effects on circulating levels of lipid-soluble EDCs. For example, a weight loss intervention among obese participants resulted in elevated levels of serum organohalogenated contaminants and their hydroxylated metabolites (96). In a study of 45 obese women who either received dietary counseling or underwent bariatric surgery, serum PCB levels were higher among those who lost weight; moreover, in the dietary intervention group, the rise in PCB levels were more pronounced among those who lost relatively more of the metabolically deleterious visceral adipose tissue (97). This finding is consistent with studies showing increases in plasma organochlorine levels proportional to the extent of weight loss (98) and increases in plasma PCBs and organochlorine pesticides after gastroplasty (99). The concentrations of POPs in breast milk is also associated with maternal weight loss (113). Bariatric surgery in 71 obese subjects resulted in significant weight loss, increased serum POP levels, and a 15% reduction in total PCB burden (100). In this study serum POP concentrations correlated with liver dysfunction although liver function and other metabolic parameters improved with weight loss (100). Another study also showed that the increase in PCB levels following weight loss did not attenuate the metabolic benefits of weight loss (114). Weight loss is known to lower basal metabolic rates and decrease levels of triiodothyronine (113, 114); whether increased PCB levels augment expected reductions in metabolic rates via known PCB-induced disruptions in the thyroid hormone axis requires further study (10). In general, these data indicate that, despite increases in blood levels of organochlorines, standard weight loss interventions remain an essential tool for addressing MDC-associated metabolic dysfunction. Moreover, weight loss-mediated mobilization of stored POPs may facilitate whole body elimination through complementary approaches, such as olestra, cholestyramine, dietary fiber, and other techniques.

### The Impact of Diet as Insight and Intervention

An increasing number of studies have begun to examine the coordinate effects of diets and MDCs on metabolic physiology. The most common hypothesis explored in these studies is that dietary parameters, most often a high fat or high fat plus high sucrose (“Western”) diet, will unmask or potentiate the adverse effects of MDCs on energy homeostasis. Developmental exposure to MDCs likely increases metabolic disease susceptibility. Thus, it is likely that a second hit, such as high fat/sugar diets or lack of exercise, will unmask latent disease susceptibility. There are now a few studies that explore the idea that MDCs add to, or synergize with, widely accepted risk factors that promote metabolic disease development. For example, female mice perinatally exposed to DDT were shown to develop glucose intolerance, hyperinsulinemia, and dyslipidemia with alterations in thermogenesis (115). In mouse models of perinatal exposure, high fat feeding amplified the adverse effects of BPA on glucose metabolism in adulthood in some (116), but not all studies (117). In a rat model, paternal exposure to BPA amplified the deleterious effects of a high fat diet on adult offspring (118). In adult exposure models, male mice exposed to BPA in the context of a high fat diet exhibited glucose intolerance with concordant impairments in skeletal muscle insulin signaling (119). Rats exposed to nonylphenol in the context of a high fat, high sucrose diet exhibited elevated blood glucose levels (120). Also, nonylphenol was shown to promote NAFLD in the context of this dietary manipulation with associated lipid accumulation and inflammatory changes (121). Chronic exposure to TCDD in the context of a high fat diet promoted weight gain with sexually dimorphic effects on fat distribution and hepatic steatosis with females exhibiting increases in liver lipid content (122). In a transgenerational study tributyltin (TBT) exposure in the F0 generation increased the sensitivity of mice in the F4 generation to a high fat diet (32). These mice exhibited normal weight gain until exposure to the high fat diet, which triggered accelerated weight gain and impaired weight loss upon switching back to a low fat diet. Finally, rats exposed gestationally and lactationally to BPA exhibited increased liver lipid accumulation and inflammation on a high fat diet after weaning (123). While some of these studies did not directly compare high fat diets (or high fat, high sucrose diets) to control diets, there is a suggestion that MDCs may augment the adverse effects of a metabolically stressful diet. A critical issue is how diets enriched in fat augment absorption of lipophilic MDCs when diet and exposures are both manipulated. In contrast, when dietary manipulation is dissociated from developmental exposure, data suggest a priming of metabolic dysfunction induced by developmental exposure to MDCs that is induced later in life. These findings broadly support standard clinical interventions to address metabolic dysfunction by restricting caloric consumption and limiting consumption of excess fats and sugars.

Interestingly, the impact of an MDC on specific metabolic outcomes may be dependent on the precise dietary intervention studied. On a standard rodent chow, exposure to the phenylsulfamide fungicide tolylfluanid promoted weight gain, increased fat mass, glucose intolerance, insulin resistance, and a disruption in circadian rhythms of feeding behavior (124). The same exposure in the context of a high fat plus high sucrose diet vs. a high sucrose diet alone yielded divergent effects on key metabolic outcomes. In the context of a high fat plus high sucrose diet, tolylfluanid did not appreciably affect weight gain, reduced adipose content modestly, but markedly exacerbated glucose intolerance with no effect on insulin sensitivity (125). In contrast, when incorporated into a high sucrose diet alone, tolylfluanid reduced weight gain while augmenting fat accumulation without any appreciable change in glucose tolerance or insulin sensitivity (125). These data strongly suggest that the impact of MDCs on metabolism may be dependent on both the dietary stressor as well as the metabolic outcome. Moreover, these data indicate that dietary sugars are modulators of MDC effects. This is important because many studies focus on high fat diets alone, even though increased sugar and refined carbohydrate intake tightly correlate with the onset of the metabolic disease pandemic in the last half century (126).

On the flip side, data suggesting that high fat, high sucrose, or Western diets potentiate the adverse metabolic effects of MDCs underscore the importance of dietary interventions in the treatment and prevention of metabolic diseases. In other words, reductions in caloric intake and dietary sugar, as well as saturated and *trans* fats, may be doubly beneficial by addressing both diet-associated disease risk as well as by reducing the impact of environmental toxicants. It will be fascinating to see whether nutritional interventions can elicit metabolic improvements among individuals with known exposures. Given the marked salutary metabolic effects of the “Mediterranean Diet” on human health (127, 128), it is essential to determine whether exposures to MDCs will affect the positive effects of this diet. In this regard, a recent 2 year randomized clinical trial that compared four common diets showed no difference in the extent of weight loss by diet type; however, participants with a higher level of PFAS had augmented weight regain, which was associated with a greater decline in basal metabolic rate during weight loss and less increase in basal metabolic rate during weight regain (129).

One exciting implication from these dietary studies is the potential to gain insights from cross-cultural comparisons of MDC effects. Comparisons of MDC effects across countries may leverage inherent differences in diets to identify those dietary components that modulate the impact of MDCs on metabolic health. Uncovering those protective dietary factors among populations less likely to suffer the adverse effects of MDCs may provide practitioners with valuable knowledge for advising patients on how to reduce their risk of MDC-induced/amplified disease.

While reducing exposure to MDCs is the primary and most effective strategy to reduce the toxic effects of these chemicals, a complimentary approach involves the activation of metabolizing systems to reduce the level of the active chemicals post-exposure (130). Specific dietary constituents may provide such an effect. *Brassica* crops (e.g., cabbage, broccoli, cauliflower, kale, collards, and brussel sprouts) contain chemicals that release sulforaphane upon hydrolysis. Sulphoraphane has been shown to help reduce obesity (131), improve glucose tolerance (132), and restore leptin sensitivity in high fat-sucrose diet fed obese mice (133). Sulforaphane functions by protecting against oxidative stress via activation of the Nrf2-ARE pathway in multiple tissues (134, 135). Also, sulforaphane has anticancer activity stemming from its effects on DNA methyltransferases and histone deacetyltransferaces as well as its epigenetic reactivation of Nrf2 (136). It is important to explore whether sulforaphane can protect against MDC action in humans. It is essential to recognize, however, that in some cases the metabolites of an MDC may be more toxic than the parent compound. One such example is benzo(*a*)pyrene. In this case, resveratrol-mediated *impairments* in benzo(*a*)pyrene metabolism were shown to reduce colon cancer incidence and tumor size in a rat model by lowering DNA adduct formation, an essential mechanism in cancer initiation (137). Thus, while dietary or pharmacological induction of MDC metabolism pathways may hold promise, more work is needed to discern which MDCs are more likely to be detoxified by such an approach.

### Merging Mechanisms of Action to Modes of Toxicity

As scientific advances in the field illuminate modes of metabolic toxicity, new opportunities will arise to individualize disease management plans. Over the course of the last decade, there have been significant advances in our pharmaceutical armamentarium for treating individuals with obesity, diabetes, NAFLD, and other metabolic conditions. Importantly, this includes entirely new classes of medications that address pathophysiological defects inherent to metabolic diseases. Recently, several signaling cascades have emerged as being critical for disease development. In diabetes, two examples are the sodium-glucose co-transporter 2 system in the kidney and gut-derived incretin hormones (33). The importance of these systems as well as our clinical capacity to modulate their activity raise three fundamental questions for the field. First, we must understand whether MDCs modulate these newly uncovered pathways. This understanding is especially salient for MDCs for which mechanisms of metabolic toxicity remain poorly delineated. Thus, emerging knowledge of disease processes should expand hypotheses regarding the modes by which MDCs induce or amplify metabolic dysfunction. Secondly, our rapidly increasing capacity to tailor medical therapy to the individual opens up unique opportunities to address the impact of known exposures. Indeed, a significant focus in diabetes care, as well as in other areas of medicine, is on the individualization of treatment (33). For patients with known exposures that cannot be meaningfully reduced or eliminated, incorporation of exposure assessment with consideration of MDC-associated mechanisms of toxicity offers another opportunity to individualize pharmacological management of their metabolic disease. For us to incorporate exposures into clinical decision making, data gaps need to be filled. There are many combinations of exposures in humans, which creates challenges to understanding the importance of MDCs as well as in devising targeted interventions. Regardless, the opportunities to leverage this knowledge have never been better. Third and finally, there are prospects for exposure science to drive drug development and disease treatment. Where environmental exposures have been linked with disease, examining metabolic toxicity using unbiased approaches may lead to new mechanisms of action that could be relevant to disease development more generally; moreover, these new pathways may be potentially druggable. In this way, MDCs may be used as molecular tools to uncover new biology that has the potential to improve human health.

### Improving Resilience During Pregnancy

In addition to interventions meant to address the epigenetic alterations induced by MDC exposure, other interventions have been shown to protect against several environmental toxicants in the context of non-metabolic diseases. For example, some fish nutrients have been posited to protect children from the deleterious neurodevelopmental effects of mercury (138). Iron deficiency augments lead absorption (139, 140), while iron-enriched diets appear to reduce transplacental transfer of lead (141). Similarly, higher dietary calcium intake and calcium supplementation during pregnancy and lactation may also minimize lead exposure of fetuses and infants (142, 143); however, this effect may be dependent on baseline calcium intake (144). In the context of arsenic exposure, several interventions have been shown to attenuate toxicity of this known MDC, including ascorbic acid and tocopherol (145–150); plants extracts, flavonoids, and polyphenols (151–154); and selenium (155–160). Finally, pregnant women instructed to take supplements of *Chlorella pyrenoidosa* (an algal extract; 6 g/day for 6 months) demonstrated 30% lower total toxic equivalents in breast milk relative to controls (161). While some of these interventions may be specific to the environmental toxicant studied, they suggest that targeted maternal interventions may reduce fetal and infant exposures resulting in a reduction in long-term, environmentally-mediated metabolic risk. Further work is needed, however, to ascertain whether these interventions meaningfully impact energy homeostasis.

## Collision of Exposure Science, Medical Ethics, and Patient Care

Importantly, the gap in our knowledge of MDC sources has a unique extension into the field of clinical medicine, namely exposures arising from medical treatments. This creates novel ethical concerns for providers. Our modern healthcare system is built upon central tenets of medical ethics that guide the provision of care to patients. These principles include respect for autonomy, beneficence, non-maleficence, and justice (162). Related and critically important extensions of these ethical concepts include patient's rights to disclosure and for medical decisions to be made through a process of informed consent. While clinicians often do an excellent job of discussing the risks and benefits of specific pharmacological therapies, rarely (if ever) discussed are the potential impacts of “inactive” ingredients included in particular drug formulations. Undoubtedly, this is partially attributable to a lack of physician awareness and knowledge; however, the consequence is that patients are not appropriately informed of treatment risks and potential alternatives, violating the central tenet of autonomy. This is relevant to the current discussion because some medications are formulated with phthalates (163–165) and other synthetic polymers (166), making iatrogenesis a potentially significant source of phthalate and additional MDC exposure. Indeed, pharmaceuticals can be a potential source of dibutyl phthalate, a compound with known adverse reproductive and developmental effects (163). The positive association between current use of polymer-containing drugs and high rates of poor semen quality in men provide further evidence for this premise (167). Iatrogenic risk can be imposed by other physician-initiated practices as well, including infusion systems. In one study, switching from DEHP-plasticized polyvinylchloride (PVC) infusions systems to PVC-free lines for total parenteral nutrition (TPN) led to a marked reduction in TPN-associated cholestasis (168). This case illustrates both the importance of unappreciated and clinically significant risk associated with some exposures as well as the profound opportunity to reduce adverse outcomes by choosing delivery systems that avoid disease-associated exposures. Importantly, it also emphasizes the need for physicians to address fundamental knowledge gaps in this realm in order to empower their patients to make informed decisions about their care.

## Conclusions

**Figure 2:**
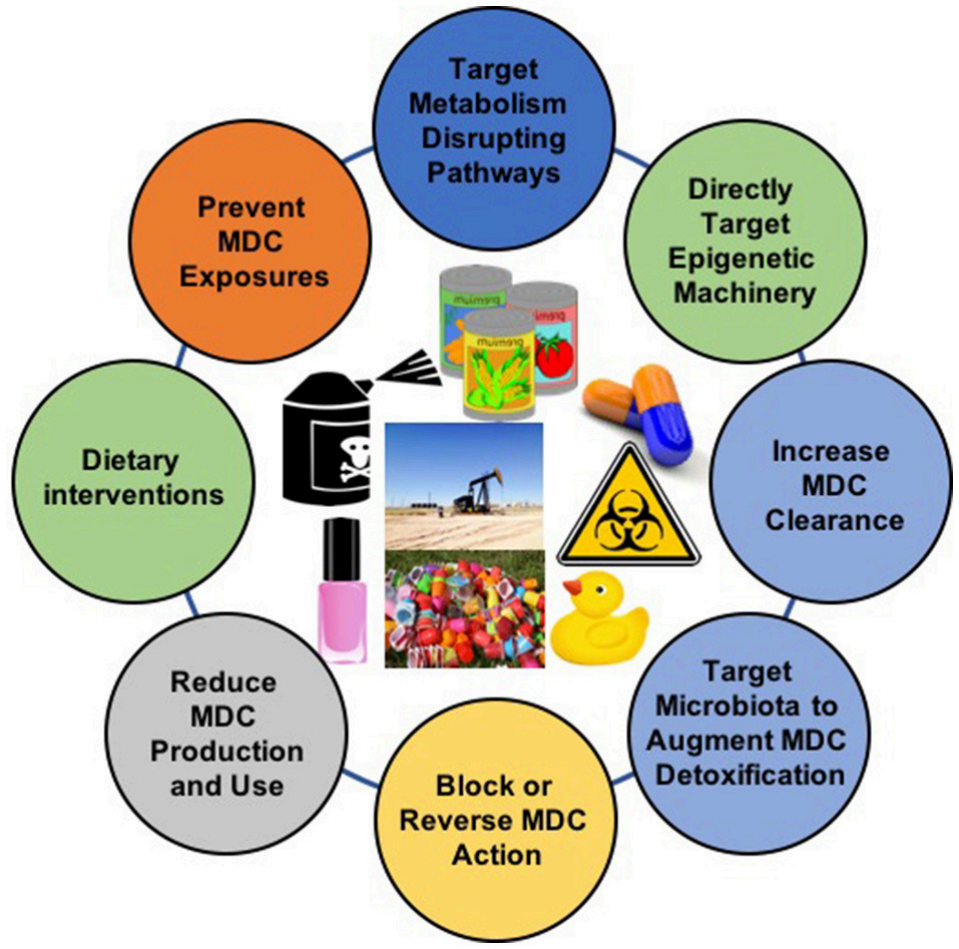
Interventions to Address Exposure to Metabolism-Disrupting Chemicals (MDCs). The diversity of MDCs and their effects require comprehensive approaches to address this underappreciated metabolic disease risk factor. Images from pixabay.com and openclipart.org.

Increasing evidence implicates MDCs as contributors to the burgeoning global pandemic of metabolic disease. Addressing these novel risk factors requires the implementation of multifactorial intervention strategies across the lifespan (Figure 2). While more data are needed, the potential benefits of these approaches are now supported by a variety of proof-of-concept studies in animal models and humans (Table 2). Given the importance of developmental programming in metabolic outcomes, efforts to reduce maternal MDC exposure, prevent transplacental and lactational transmission of MDCs, and inhibit deleterious epigenetic alterations induced by MDCs offer unique opportunities to impact health across generations. The best studies in this area focus on interventions to reduce maternal-fetal transmission of specific MDCs, such as lead. Whether similar approaches will work for other common MDCs remains to be determined. Efforts to address metabolic programming through manipulation of one-carbon metabolism in order to affect DNA methylation or interventions to reverse altered programming *per se* may hold promise, but fundamental questions remain regarding whether such interventions are sufficiently specific to address MDC action while minimizing off-target epigenetic programming events. Resolving these challenges is critical as interventions targeting pregnancy and early life development have the potential to reduce long-term disease susceptibility, potentially across generations.

**Table 2:** Potential intervention strategies to reduce the deleterious effects of metabolism-disrupting chemicals.

**Strategy**	**Chemical class(es)**	**Interventions**	**Models**	**Reference(s)**
Employ existing resources that have compiled exposure sources and modify individual behaviors to reduce contact with those chemicals.	Various	Various	Humans (proposed)	(11, 36–47)
Identify and discontinue behaviors shown to *increase* exposure	Phthalates	Reducing fast food consumption	Humans	(48, 49)
	Bisphenol A	Avoiding use of polycarbonate water bottles	Humans	(50)
	Bisphenol A	Reducing canned soup consumption	Humans	(51)
	Air pollutants	Reducing contact with polluted urban air	Humans	(55–57)
Adopt practices shown to *decrease* exposures	Phthalates, parabens, triclosan, and benzophenone	Reducing use of personal care products	Humans	(52)
	Bisphenol A and phthalates	Consuming foods with limited packaging	Humans	(53)
	Phthalates	Washing hands and reducing use of plastic cups for beverages	Humans	(54)
Employ supplements to mitigate the adverse programming effects of exposures during development*	Bisphenol A	Supplement diets with methyl donors, including betaine, choline, folic acid, and vitamin B12	Rodents	(60)
	Bisphenol A	Maternal folate intake	Humans	(61)
	Arsenic	Maternal folate and B12 supplementation	Rodents	(63)
Discontinue supplements shown to potentiate the adverse developmental effects of MDCs*	Arsenic	Maternal folate supplementation	Rodents	(62)
Employ supplements that antagonize the mechanisms of MDC action	PCBs	Supplement with resveratrol	Rodents	(64)
	PCBs, arsenic	Supplement with N-acetylcysteine	Rodents	(65, 66, 69)
	Arsenic	Supplement with N-acetylcysteine and monoisoamyl dimercaptosuccinic acid	Rodents	(68, 70)
	Arsenic	Supplement with N-acetylcysteine + zinc	Rodents	(71)
Consume healthful diets	PCBs	Diets rich in fruits and vegetables	Humans	(73)
	PCBs	Green tea-containing diets	Rodents	(76)
	Benzene and acrolein	Broccoli sprout beverage containing glucoraphanin and sulforaphane	Humans	(77)
Facilitate excretion of persistent organic pollutants	TCDD, PCBs, β-hexachlorocyclohexane	Treatment with the non-digestible fat olestra (Olean™)	Humans	(93–95, 101)
	PCBs, DDT, DDE	Supplementation with 1,000 mg/day ascorbic acid (vitamin D)	Humans	(102)
	PCBs	Treatment with cholestyramine	Rodents	(104)
	PCBs and polychlorinated dibenzofuran	Treatment with cholestyramine	Humans	(105)
	PCBs	Diets supplemented with chitosan	Rodents	(108)
	PCBs	Diets enriched in fermentable fibers (polydextrose, indigestible dextrin, and soy polysaccharides)	Rodents	(108)
	Polychlorinated dibenzo-*p*-dioxins, Polychlorinated dibenzofurans	Consumption of fermented brown rice with *Aspergillus oryzae* after each meal	Humans	(109)
	PCBs	Consumption of wheat bran-enriched diets	Rodents	(111, 112)
Avoid diets that amplify the adverse effects of MDCs	DDT, BPA	Perinatal exposure coupled with later-life high fat diet	Rodents	(115, 116, 118, 123)
	BPA, nonylphenol, TCDD	Concurrent exposure with high fat or high fat-high sucrose diet	Rodents	(119–122)
	TBT	TBT exposure in F0 generation with high fat diet in F4 generation	Rodents	(32)
	Tolylfluanid	Concurrent exposure to tolylfluanid and either a high fat-high sucrose diet or a high sucrose diet alone	Rodents	(125)
Employ supplements that impair the activating metabolism of toxicants	Benzo(a)pyrene	Administration of resveratrol impaired benzo(a)pyrene metabolism and markers of colon cancer	Rodents	(137)
Address vitamin and mineral deficiencies that augment exposure to environmental toxicants	Mercury	Nutrients from fish are associated with protection against neurotoxicity	Humans	(138)
	Lead	Iron deficiency augments lead absorption while iron-enriched diets reduce transplacental transfer of lead	Humans	(139–141)
	Lead	Higher calcium intake and calcium supplementation reduces exposure among fetuses and infants	Humans	(142–144)
Consider supplementation with dietary adjuncts that antagonize mechanisms of toxicity in non-metabolic models that may also be related to metabolic disruption	Arsenic	Various interventions employing ascorbic acid and tocopherol; plant extracts; flavonoids and polyphenols; and selenium	Cell-Based, Rodents, and Humans	(147–162)
Employ supplements that reduce transmission of toxicants via breast milk	Dioxins	Supplementation with *Chlorella pyrenoidosa* extract during pregnancy reduced total toxic equivalents in breast milk	Humans	(161)

* *Denotes an area in which both beneficial and harmful outcomes have been reported for the same intervention*.

Importantly, many of the same interventions to reduce levels of MDCs and their adverse effects during pregnancy are useful across the lifespan. These include behavioral efforts to reduce exposures to known MDCs and providing consumers with the information essential for making informed choices. Indeed, these are likely the types of interventions that are most likely to have a clinically significant impact, especially for those MDCs that are non-persistent. Additionally, efforts to specifically antagonize the mechanisms by which MDCs induce metabolic dysfunction have shown some promise; however, this area needs more work. Unfortunately, some of the most comprehensively characterized MDCs are persistent pollutants with long biological half-lives that are less amenable to behavioral interventions. Thus, addressing these MDCs is especially challenging; however, some evidence suggests that disrupting the enterohepatic circulation of lipophilic MDCs may lead to meaningful reductions in the overall body burden of these toxicants. Unfortunately, most data exist for treatments with relatively noxious adverse effects; therefore, alternative approaches are needed for facilitating elimination of POPs. Where known exposures are linked to clear molecular mechanisms of metabolic dysfunction, use of pharmacological therapies specifically targeting MDC mechanisms of action may allow individualized treatment regimens that address the precise origins of a patient's metabolic dysfunction. Finally, avoidance of diets that amplify the deleterious effects of MDCs in favor of those that antagonize their effects is essential, while standard clinical advice to exercise and lose weight are equally necessary components for addressing the threat of environmental toxicants to metabolic health. As we gain a greater appreciation for how the diverse array of intrinsic and extrinsic risk factors that promote metabolic disease development interact with each other (27, 169), new interventions are likely to emerge.

In the end, however, any effective strategy must include comprehensive and sustained efforts to reduce exposures wherever and whenever possible. Current federal environmental policies do not account for metabolic disease risk, as recently discussed for diabetes (170). Incorporating metabolic health as a relevant outcome in local, regional, national, and international policy has the potential to transform risk assessment to support legislation that is likely to reduce exposures. In the end, reducing exposures is at the core of any effective intervention to address the adverse effects of a toxic environment on our health. To realize these preventive strategies, we need to change the narrative from a focus on managing chronic diseases to eliminating those factors that drive disease pathogenesis, including exposure to MDCs. To do so, clinicians must begin to appreciate the contribution of environmental toxicants to metabolic disease risk and begin to incorporate this knowledge into clinical practice. In the end, however, meaningful change on this front must occur at all levels from individuals to families, to clinicians, and ultimately to policy makers. The time to begin these efforts is now as the benefits of such an approach for both the individual and society are potentially enormous.

## Disclosure

RS declares he has received honoraria from CVS/Health and American Medical Forum.

## Author Contributions

RS and VP conceived the manuscript. RS, JH, and VP all wrote and edited the manuscript.

### Conflict of Interest Statement

The authors declare that the research was conducted in the absence of any commercial or financial relationships that could be construed as a potential conflict of interest.
